# The safety, benefits and future development of overnight and outpatient thyroidectomy

**DOI:** 10.3389/fendo.2023.1110038

**Published:** 2023-04-05

**Authors:** Duntao Su, Zeyu Zhang, Fada Xia, Xinying Li

**Affiliations:** ^1^ Department of General Surgery, Xiangya Hospital, Central South University, Changsha, Hunan, China; ^2^ National Clinical Research Center for Geriatric Disorders, Xiangya Hospital, Central South University, Changsha, Hunan, China

**Keywords:** overnight thyroidectomy, outpatient thyroidectomy, postoperative complication, economic benefit, mental healthy

## Abstract

With the development of medical care, the safety of thyroidectomy is improving year by year. Due to economic benefits and other advantages of the overnight and outpatient thyroidectomy, more and more patients and medical institutions have favored overnight and outpatient thyroidectomy, and its proportion in thyroidectomy has increased year by year. However, overnight and outpatient thyroidectomy still faces many challenges and remains to be improved. In this review, we focused on the recent progress and the relevant clinical features of overnight and outpatient thyroidectomy, including its safety, economic benefits, etc., which may bring valuable clues and information for further improvements of patient benefits and promotions of overnight or outpatient thyroidectomy in the future.

## Introduction

Thyroid nodule is one of the most common diseases of the endocrine system. Studies have shown that the incidence of thyroid nodules is 19%-67% nowadays. Among them, thyroid cancer accounts for about 5%, of which more than 90% are papillary thyroid carcinoma. Thyroid cancer is expected to become the fourth cancer in the world in terms of incidence rate, especially among women. From 1990 to 2013, the world’s age-standardized incidence rate of thyroid cancer increased by 20% (1). From 1990 to 2017, with the continuous progress of ultrasound technology and equipment, the incidence rate and mortality of thyroid cancer increased year by year (2). According to the global cancer statistics in 2020, the newly diagnosed cases of thyroid cancer reached 586202, and the global death toll was 43646 (3). Thyroidectomy takes a very important part in the treatment of thyroid cancer. If active surgical intervention is adopted, early-stage patients can have a better prognosis with a five-year survival of 98% (4, 5). With the improvement of thyroidectomy technology and safety in recent years, overnight and outpatient thyroidectomy have gradually become popular.

Outpatient thyroidectomy was first mentioned in 1986. Due to its economic benefits and other advantages, more and more patients and medical institutions have favored overnight and outpatient thyroidectomy, and its proportion in thyroidectomy has increased year by year (6, 7). Outpatient thyroidectomy means that the patients are discharged on the same day after their vital signs are stable to return to their homes for rest and recovery. Overnight thyroidectomy was first reported in the literature in 2004 (8). The most significant difference between overnight thyroidectomy and outpatient thyroidectomy is whether you will spend one night in the hospital after surgery. The patients are often discharged from the hospital the next morning after the overnight thyroidectomy and return to the residence for observation. On the one hand, overnight and outpatient thyroidectomy have certain economic and time benefits. On the other hand, due to the short postoperative observation time, postoperative complications are important factors affecting postoperative safety, including hypocalcemia, neck hemorrhage, recurrent laryngeal nerve injury, seroma, etc. (9–12). Moreover, the selections of patients and the surgical approaches are still controversial in overnight and outpatient thyroidectomy.

In this review, we focused on the recent progress and the relevant clinical features of overnight and outpatient thyroidectomy, including its safety, economic benefits, etc., which may bring valuable clues and information for further improvements of patient benefits and universalization of overnight or outpatient thyroidectomy in the future.

## Overnight thyroidectomy

Overnight thyroidectomy is defined as the patients being observed in the medical institution overnight in postoperation and discharged within 24 hours. Compared with inpatient patients, patients undergoing overnight thyroidectomy have a shorter hospital stay and economic benefits. Although most of the studies showed that there was no statistical difference in the safety between inpatient and overnight patients, certain problems existed due to the short observation time (13). In this review, we mainly focused on the recent progress of overnight thyroidectomy in the last decade.

## Progresses in surgical approaches

Overnight open thyroidectomy has become mature. In recent years, increasing studies have begun to try some surgical approaches different from open surgery in overnight thyroidectomy to meet the growing need for cosmesis. A French study was the first to try transoral endoscopic thyroidectomy *via* vestibular approach (TOETVA) in overnight thyroidectomy. A total of 374 patients were recruited and divided into a TOETVA group and an open surgery group. There was no difference in physical and mental scores of QoL or postoperative complications compared with inpatient surgery (14). Zhang et al. explored the differences in safety and economic benefits between endoscopic thyroidectomy *via* a chest-breast approach (ETCBA) and routine inpatient ETCBA. The study included 260 patients, of whom 206 (79.2%) is thyroid cancer. It is as safe as patients with benign thyroid nodules or thyroid cancer, with significant economic benefits and shorter hospital stays. Particular attention should be paid to the relief of anxiety and stress in patients receiving overnight ETCBA (15). The details of the two studies are shown in **Table 1**. With the continuous advancement of medical technology, we believe that increasing surgical approaches will appear in overnight thyroidectomy, such as transaxillary thyroidectomy and retroauricular thyroidectomy.

**Table 1:** The details of two endoscopic studies.

Study	Country	Year	Patient	Type	Approach
Zhang et al. (15)	China	2021	260	Retrospective stydy	ETCBA
K. Van Den et al. (14)	French	2022	374	Prospective stydy	TOETVA

## Patient selection and preoperative preparation

### Patient selection

Since the observation time of overnight thyroidectomy is shorter than that of inpatient, not all patients are suitable for overnight surgery, and more caution is needed in selecting patients. The inclusion criteria are (a) Patients under 60 years old (b) First thyroidectomy (c)No cervical lateral lymph node metastasis or distant metastasis in preoperative examinations. Exclusion criteria are generally (a) Thyroid gland external tissue invasion, such as muscle, etc. (b) American Society of Anesthesiology (ASA) grade III or higher (c) Severe chronic diseases such as severe liver and kidney insufficiency (d) Currently taking oral anticoagulation or antiplatelet drugs (13–20). There are also subtle differences in preoperative patient selection between different surgical approaches, with TOETVA requiring thyroid nodules volume<45 ml and/or nodule diameter<50 mm. Cytological examination Bethesda grade less than grade 4, or nodules less than 2 cm in the case of Bethesda grade ≥ 5, and no lateral lymph node spread or mediastinal metastasis (14). The exclusion criteria for patients with ETCBA were lateral lymph node metastasis (15). Different from the impatient surgery, the location of the post-operative recovery of the patients in the overnight thyroidectomy is close to the medical institution, which is also a factor to be considered.

### Preoperative preparation

In addition to routine preoperative preparation for surgery, all patients underwent preoperative neck ultrasound to assess thyroid nodule size and lymph nodes before surgery. Some medical institutions will perform ultrasound-guided fine-needle aspiration and pathological classification of patients with suspicious nodules (14, 15). Different approaches of overnight endoscopic thyroidectomy have different requirements for preoperative preparation. For example, patients undergoing TOETVA surgery are treated with antibiotics for five consecutive days during the perioperative period, and Chlorhexidine is used to clean the mouth 3 times a day, starting the day before the surgery and continuing for at least 5 days (14). Careful and comprehensive preoperative examination combined with preoperative preparation is an important measure to ensure the safety of patients undergoing overnight thyroidectomy.

## The associated complications

### Anesthesia-related complications

Most of the drug deaths in the United States in 2015 were related to opioids, and a study showed that Multimodal analgesia (MMA) can be used safely in neck surgery and can reduce the use of prescription opioids. Opioids are still the primary means of managing chronic and acute pain, and their use may increase drug-related adverse effects and the risk of addiction and abuse. At present, anesthesiologists should choose a more scientific and healthy multimodal analgesia. On the one hand, it can reduce the occurrence of postoperative nausea and vomiting. On the other hand, non-steroidal drugs should be used more rationally. Some studies have found that if careful intraoperative hemostasis is combined with perioperative use of NSAIDs as part of an MMA regimen did not increase the risk of postoperative hemorrhage (21).

Postoperative nausea and vomiting (PONV) are common adverse reactions after overnight thyroidectomy. To improve the comfort of patients, anesthesiologists can effectively reduce the incidence of postoperative PONV by rationally selecting drugs and anesthesia methods during the operation. Studies have shown that moderate use of dexmedetomidine in thyroidectomy can reduce the use of postoperative anesthesia, which will help patients’ postoperative analgesia (22, 23). However, the use of dexmedetomidine during overnight thyroidectomy did not reduce the incidence of PONV at 24 hours in postoperation (p=0.355) (24). To reduce the incidence of the postoperative POVN, intraoperative cervical plexus anesthesia block, rational use of anesthetic drugs in preoperative multimodality, postoperative use of non-steroidal drugs such as Acetaminophen or Ibuprofen in combination with appropriate Opioid analgesia has a good effect on preventing postoperative POVN, which can also indirectly reduce the dosage of opioids (25). Therefore, anesthesiologists should consider more factors in the choice of anesthesia drugs and anesthesia methods and choose the types of drugs and anesthesia methods more carefully.

### Postoperative hemorrhage

Postoperative hemorrhage after thyroidectomy is one of the most dangerous complications after thyroidectomy. The probability of hemorrhage after thyroidectomy is about 0.07%-4.2% (11, 26, 27). Risk factors for hemorrhage after thyroidectomy include older age, male gender, larger thyroid nodule, current hemorrhage, current oral anticoagulant or antiplatelet medication, and inexperienced surgeon (28).

With the rapid development of energy devices, there are more and more intraoperative hemostasis techniques available during overnight thyroidectomy and it can further reduce the risk of postoperative hemorrhage in patients. The most advanced energy technology such as ultrasonic devices or bipolar electrocautery is widely used in surgery now. But the most important thing to prevent hemorrhage after thyroidectomy is the surgeon’s careful hemostasis and firm ligation of every blood vessel during the operation. In addition, there are a variety of other measures to reduce the risk of life-threatening postoperative hemorrhage. Previous studies have suggested that dyspnea after thyroidectomy is related to the short-term accumulation of blood or lymphatic fluid in the surgical area, resulting in a short-term increase in local pressure, thereby compressing the airway and causing dyspnea (29). Some doctors believe that suturing only part of the strap muscles or even not suturing the strap muscles during the operation can effectively reduce the risk of suffocation because there is enough space to release the pressure when the neck hemorrhage are happening so it can effectively delay the time of compressing the trachea (30). In terms of the use of biomaterials, the use of biomaterials such as the Collagen-Fibrinogen-Thrombin Patch in the intraoperative wound area can also effectively reduce the risk of exudation and hemorrhage (31).

There is no statistically significant difference in the incidence of postoperative hemorrhage between overnight thyroidectomy and inpatient thyroidectomy in terms of the incidence of postoperative hemorrhage (17). The difference in hypoparathyroidism in ECTBA was also not statistically significant (15). A prospective randomized controlled trial of 411 patients showed no difference in the incidence of complications such as hypoparathyroidism, hoarseness, hematoma, and wound infection between overnight and inpatient thyroidectomy (16). Although hemorrhage after thyroidectomy can occur at any time, most hemorrhage occurs within 24 hours in postoperation (32, 33). Therefore, most of the hemorrhage after overnight thyroidectomy occurs in the hospital, which means almost all hemorrhage can be detected in time (19). People in developing countries may have a shorter chance of receiving proper complications treatments due to the lower primary care and lower density of medical institutions than in developed countries. Furthermore, the poor and congested traffic can also be an important hindrance for patients to the medical institution on time in developing countries. Finally, the identification and emergency management of complications are also critical for patients and their caregivers, so it is necessary to train patients and their families about complications.

### Hypoparathyroidism

Hypoparathyroidism is one of the most common complications after thyroidectomy. Hypoparathyroidism after overnight thyroidectomy is a crucial factor affecting the discharge of patients within the prescribed time. The meticulous anatomy of the parathyroid glands and the protection of the blood supply of the parathyroid glands during surgery are effective measures to prevent postoperative hypoparathyroidism. In addition, intraoperative parathyroid autotransplantation can effectively prevent most permanent hypoparathyroidism, so each parathyroid gland should be protected as the last parathyroid gland during surgery (34). At present, many studies have predicted postoperative hypoparathyroidism and recovery by measuring the results of PTH or serum ions at different time points. These methods include the detection of serum ions or PTH levels in serum before, during, and after surgery, which can predict whether permanent or transient hypoparathyroidism will occur in postoperation (35). Hypoparathyroidism is manifested as hypocalcemia, mild paresthesia, severe tetany, and a series of neuromuscular symptoms. In a randomized controlled trial of 411 patients from China, Zhang et al. showed that the incidence of either hypoparathyroidism after overnight thyroidectomy was not statistically significant compared with hospitalized patients(P =0.631) (16). Another study found that the incidence of hypoparathyroidism even in overnight thyroidectomy patients was lower than that in inpatient patients (9.26% vs 14.3%; P< 0.05), which may be related to the prudent choice of overnight thyroidectomy patients before surgery (17). The difference in hypoparathyroidism in ECTBA was also not have statistically significant (15). Overall, overnight thyroidectomy was not significantly different or less frequent than inpatient thyroidectomy in terms of the incidence of hypoparathyroidism or hypocalcemia.

At the same time, the publicity and education of patients and their caregivers should also be done well. An postoperative active calcium supplementation strategy is beneficial to the relief of postoperative hypoparathyroidism symptoms and the recovery of parathyroid function (36). When a patient is discharged from the hospital and develops severe hypoparathyroidism at home, such as cramps in the hands and feet, and a prickling sensation around the mouth, that cannot be relieved by self-administered oral calcium supplementation. Patients should be able to get calcium supplements to the nearest healthcare facility in time to avoid life-threatening consequences due to low calcium levels.

### Recurrent laryngeal nerve injury

Although the rate of recurrent laryngeal nerve (RLN) injury during thyroidectomy is low, bilateral recurrent laryngeal nerve palsy is one of the most serious complications after thyroidectomy. When one recurrent laryngeal nerve injury can cause hoarseness, and when bilateral recurrent laryngeal nerve injury can lead to airway obstruction and life-threatening, emergency tracheal intubation or tracheotomy is needed. There was no significant difference in the incidence of recurrent laryngeal nerve injury during Overnight thyroidectomy and postoperative hoarseness. In this study of 870 patients, the incidence of recurrent laryngeal nerve injury was approximately 4%, and there was no statistical difference between overnight and inpatient patients (p=0.188) (17). The incidence of complications was also not statistically different in patients with ETCBA (15). Intraoperative nerve monitoring technology can effectively detect the relevant recurrent laryngeal nerve injury and effectively reduce the risk of recurrent laryngeal nerve injury during surgery. If intraoperative neuromonitoring has found damage to one RLN, it can effectively remind the surgeon to be more careful when dealing with the contralateral RLN to reduce the risk of bilateral RLN injury (37, 38). More importantly, if the recurrent laryngeal nerve injury can be detected in time during the operation, the patient’s vital signs (such as respiratory indicators) can be detected more specifically, and the observation time can be extended, or hospitalization can be selected in the post-surgery period to reduce possible life-threatening risks.

## The economic benefits and patient satisfaction

The advantages of overnight thyroidectomy, such as shorter hospital stays, are not the real motivation for patients to choose it. Economic factors are often the strong motivation for patients to choose overnight surgery. Overnight thyroidectomy is more economical than inpatient thyroidectomy due to the shorter hospital stay. A randomized controlled study by Zhang et al. found a savings of about one-third in the cost of overnight thyroidectomy compared to inpatient thyroidectomy (16). Even in ETCBA surgery, overnight thyroidectomy still had a greater economic advantage over inpatient thyroidectomy (20568.27 ± 4476.33-yuan vs 14778.09 ± 2092.45-yuan p<0.001). A study from the United Kingdom showed a significant reduction in the cost of hospitalization for overnight thyroidectomy ($7158 vs $9525; P<0.001), and the difference in mean financial burden between patients undergoing overnight thyroidectomy and inpatient thyroidectomy was $2367 (17). Linen Mao et al. also came to the same conclusion (13). More importantly, the rates of complications were not statistically significant in any of the four studies between overnight and hospitalization thyroidectomy. The specific cost data can be seen in **Table 2**.

**Table 2:** The cost data of inpatient and overnight thyroidectomy.

Study	Country	Year	Patient	Type	Inpatient cost	Overnight cost	*p*
Zhang et al. (16)	China	2021	411	Radom trial stydy	17978.77 ± 1737.12yuan	13323.31 ± 952.85 yuan	<0.01
Zhang et al. (15)	China	2021	260	Retrospective stydy	20568.27 ± 4476.33 yuan	14778.09 ± 2092.45 yuan	<0.001
Linfeng Mao et al. (13)	China	2016	66	Retrospective stydy	17418.40 ± 2617.20 yuan	11645.04 ± 2187.01 yuan	<0.01
P. Rosen et al. (17)	British	2021	870	Retrospective stydy	$9525	$7158	<0.01

Fewer hospitalization costs are not only an important factor in patient satisfaction, which also includes changes in patients’ quality of life after surgery, more convenient medical facilities, and the comfort and convenience of patient’s recovery at home (which requires patients to receive effective education before discharge) (20). Post-thyroidectomy complication rates are relatively low, and overnight thyroidectomy has less impact on gastrointestinal function and activity than more invasive overnight surgery involving abdominal viscera or joints. Patients can eat or get out of bed within a brief period of postoperation, and they can usually control postoperative pain better. Studies have shown that whether it is benign thyroid nodules or differentiated thyroid cancer, the satisfaction score of overnight thyroidectomy is significantly higher than that of inpatient thyroidectomy (p<0.01) (13). In terms of the postoperative mental health of patients, the Depression Anxiety Stress Scales-21(DASS-21) scale was used to measure the postoperative mental health score of patients. The study found that there was no statistical significance between the endoscopic and open surgical approaches in depression (p=0.758) and anxiety (p=0.390). However, the stress of overnight thyroidectomy patients was significantly higher than that of inpatient patients (p<0.001) (16). Almost the same conclusion was drawn for patients receiving endoscopic thyroidectomy There was no statistical difference in the quality of life (p=0.28) or mental (p=0.569) of patients who underwent overnight or inpatient TOETVA overnight thyroidectomy (14). However, in the ECTBA approach, it was no significant difference in depression between overnight and inpatients thyroidectomy, but anxiety and stress were significantly higher than in inpatients, which may be because the authors did not count the mental state of the patients before participating in the study (15).

Patients who choose overnight thyroidectomy can significantly reduce the economic burden and may also benefit from the shorter hospital stay. Rehabilitation of patients in a familiar place is conducive to improving patient satisfaction. A considerable number of patients are willing to recover in a familiar family environment and the company of their family members, and thus, can obtain sufficient family support. Meantime, medical institutions may also benefit from overnight thyroidectomy because the shorter hospital stay can increase the turnover rate of the hospital and effectively reduce the management cost of medical institutions. Furthermore, the risk of hospital-acquired infection is reduced due to the shorter hospital stays, which is more in line with the current COVID-19 prevention and control strategy. However, it must be noted that patient selection is the crucial part in performing overnight thyroidectomy to ensure patient safety, which should be primarily considered before any economic and non-economic benefits.

## Outpatient thyroidectomy

Outpatient thyroidectomy is defined as discharge in post-operative 6 hours instead of overnight when the patient’s condition is stable. Many studies have shown that if reasonable screening is performed after thyroidectomy, the safety of outpatient thyroidectomy is not statistically different from inpatient surgery (11, 26, 27, 39, 40). A study of 81,199 patients showed a lower incidence of neck hematoma after outpatient surgery because of the properly selected patients, and postoperative mortality is independent of outpatient thyroidectomy (12). The same American College of Surgeons National Surgical Quality Improvement Project (NSQIP) database study found that outpatient thyroidectomy patients had lower readmission rates because the medical institutions followed the ATA guidelines for the appropriate selection of outpatient thyroidectomy patients (41). The meta-analysis of 10 observational studies also came to the same conclusion that the safety of outpatient thyroidectomy was not significantly different from inpatient thyroidectomy when selecting patients suitable for outpatient thyroidectomy (42). A safe, cost-effective outpatient thyroidectomy program can be successfully developed with appropriate patient education, in conjunction with the hospital setting and patient situation (43). However, there are still certain problems with outpatient thyroidectomy because of the short hospitalization time. In this review, new developments in outpatient thyroidectomy in the last decade have been brought to our attention.

## Patient selection and preoperative consideration

### Patient selection

Age is not a determining factor preventing patients and physicians from choosing outpatient thyroidectomy (44). At present, outpatient thyroidectomy is known to be safe and effective in adults, but few studies focus on children and adolescents. Research has found that intraoperative PTH testing and active blood serum ionization are helpful in early childhood safety discharge from the hospital (45). Studies have shown that in high-volume surgeons and high-volume medical centers, the safety and efficacy of thyroidectomy in pediatric patients are the same as in inpatient patients (46). Even among the elderly, surgeons are increasingly opting for outpatient thyroidectomy because it is as safe as inpatient thyroidectomy. However, the proper selection of patients is crucially important such as the medics should ask elderly patients about other diseases they suffer from at the same time and to take measures for complications (9, 47).

Graves’ disease thyroidectomy was previously considered more challenging than normal thyroidectomy because the thyroids with Graves’ disease are more vascularized and enlarged due to the inflammation, which will increase the risk of postoperative neck hematoma (43). In addition, the thyroid storm may happen due to improper intraoperative operation and insufficient preoperative preparation in Graves’ disease patients (48). However, recent studies have shown that surgical treatment of Graves’ disease is also safe in outpatient thyroidectomy centers if strict patients are selected and experienced (high-volume) surgeons and dense postoperative monitoring of complications (49).

What is more, the exclusion criteria for outpatient thyroidectomy also deserve our attention. An NSQIP-based study with 76,604 samples showed that although there was no difference in postoperative complications between outpatient and inpatient thyroidectomy, the study set strict exclusion criteria for patient selection. Patients with serious chronic diseases such as unexplained weight loss, long-term use of steroids, ongoing dialysis treatment, severe hemorrhage or on anticoagulant or antiplatelet therapy, and anesthesia grades III or IV should not be candidates for outpatient thyroidectomy, which will increase patient readmissions probability (50).

Furthermore, the distance between the patient home and available medical center should also be taken into consideration. For patients who need more than 2 hours to arrive at the hospital, outpatient thyroidectomy needs to be carefully considered. Once the patient has dangerous complications at home, it may be life-threatening because the distance is too far to rescue in time. Therefore, for those preparations for patients undergoing outpatient thyroidectomy but far from the hospital, it is most appropriate to stay in a hotel close to the hospital for the first night in postoperation (51–53). After returning to the residence, the patient or their caregivers must be able to correctly identify the postoperative complications, contact the surgical team at a time when some serious complications occur, and rush to the nearest medical institution for correct treatment in the shortest possible time.

In conclusion, the exclusion criteria for patients selected for outpatient thyroidectomy may include (a) Patients with serious chronic diseases. (b) The patient’s basic physical condition is too poor to stand the thyroidectomy. (c) Have severe hemorrhagic disease or undergoing anticoagulant or antiplatelet therapy. (d) Anesthesia grades III or IV. (e) The distance is less than 2 hours to the nearest medical institution. It should be considered in patient selection and interoperation.

### The extent of surgery and the selection of surgeons

The proper extent of surgery is also important for the patient who will undergo outpatient thyroidectomy. At present, the surgical scope of outpatient thyroidectomy is mainly unilateral thyroidectomy, but bilateral thyroidectomy still accounts for a small proportion (9, 10, 34). Patients with malignancies will additionally undergo cervical neck dissection. The safety of bilateral thyroidectomy and cervical neck dissection need to be further confirmed in future studies. The greater the scope of the patient’s surgery, the higher the probability of postoperative complications (54). So, doctors should make the right choice between the scope of thyroidectomy and the incidence of complications.

In a recent study of thyroidectomy patients in the Health Care Utilization Project Nationwide Inpatient Sample (HCUP-NIS), surgeons were categorized as having low volume (fewer than 10 cases annually; 6072 surgeons), intermediate volume (10-100 cases annually; 11,544 surgeons), and high volume (more than 100 cases annually; 4009 surgeons) (55). High-volume surgeons are deemed to have better surgical skills and lower complication rates and are most beneficial to the patient’s economic burden (56). However, not all doctors are high-volume surgeons. Even for moderate-volume surgeons, the rate of readmissions and complications after outpatient thyroidectomy were not had statistically significant differences (57). On the other hand, even in low-volume hospitals, a study found that the incidence of complication was not significant different with proper patient selection (56). Therefore, the success of overnight surgery is not related to the experience of the surgeon or the size of the hospital if the medical center has good patient selection guidelines, according to the above studies.

## Complications associated with outpatient thyroidectomy

Although outpatient thyroidectomy has the same complications as overnight surgery, the ever-shorter observation time in the hospital for outpatient thyroidectomy patients contributed to the focus of medical monitoring to be on the prevention of complications during the entire perioperative period and the sensitive identification of complications by patients and caregivers after returning home. Many studies have shown that outpatient thyroidectomy and inpatient thyroidectomy have no statistical difference in the incidence of various complications (10, 11, 26, 27).

### Anesthesia-related complications

The abuse of analgesic drugs has become a serious social problem, but how to control pain in outpatient thyroidectomy patients is still a question worth discussing. Based on the statistics of oral morphine equivalents (OMEQ) after outpatient thyroidectomy in two large medical institutions, it was found that approximately 93% of patients after thyroidectomy required 20 or fewer units of OMER for postoperative analgesia. Surgical patients may be allowed to prescribe an OMER of 20 units or less on an outpatient basis to help patients with effective postoperative analgesia when they are discharged from the hospital (58). To avoid the abuse of opioids and ensure their safety, the study found that postoperative use of non-steroidal drugs did not increase the risk of hemorrhage and hematoma after head and neck surgery (59). Patients cannot have specialized medical staff to control pain like inpatient surgery after outpatient thyroidectomy patients return home. Therefore, multimodal medication after thyroidectomy can reduce opioid use and minimize the risk of opioid addiction and abuse under the premise of ensuring patient comfort.

### Postoperative hemorrhage

Several studies have shown that there is no statistical difference in the incidence of postoperative hemorrhage between outpatient thyroidectomy and inpatient thyroidectomy (11, 26, 27). Thyroidectomy postoperative hemorrhage can occur at any time in postoperation. Many studies showed that approximately 50% postoperative hemorrhage occurred within 6 hours of surgery, however there was still a large proportion of hemorrhage occurred after 6 hours, which can also be lethal to patients (28, 32, 60, 61). However, since the patient is usually discharged from the hospital at this time, whether the patient can receive timely and correct treatment depends on the patient and his family to identify the hemorrhage and send him to the hospital in time (32, 33). So, it is also crucially important to train family members or caregivers to identify and deal with the hemorrhage in time, and if possible, the training of opening the incision.

### Hypoparathyroidism

The incidence of hypoparathyroidism in outpatient surgery was not statistically different from inpatients (9, 10, 34). The symptoms of hypoparathyroidism may not usually appear at once after surgery and may even appear on the 2nd or 3rd postoperative day. Therefore, most patients with hypoparathyroidism have symptoms after discharge (62). Furthermore, active calcium supplementation strategies and correct identification of hypoparathyroidism in outpatient thyroidectomy patients after discharge become critical. Some studies suggest that outpatient thyroidectomy patients should take a more active calcium supplementation program to prevent the symptoms of hypoparathyroidism after thyroidectomy. Presently, the common calcium supplementation program is oral calcium supplementation and oral calcitriol to promote calcium absorption. When the serum calcium and PTH are normal, the patient can gradually reduce the dose on their own (51, 63).

### Recurrent laryngeal nerve injury

Injury of the RLN during thyroidectomy is a common postoperative complication. The most intuitive manifestation is hoarseness, but most of them recover in a short period of postoperative. The study found that the incidence of RLN injury in outpatient thyroidectomy was not statistically different from that in hospitalized patients (p=0.17) (27). A study of 382 inpatients and 628 outpatient thyroidectomies found no significant difference in the incidence of recurrent laryngeal nerve injury within 30 days after surgery (41). These studies once again show that outpatient thyroidectomy is safe in terms of RLN protection.

### Economic benefits and patient satisfaction of outpatient thyroidectomy

Outpatient thyroidectomies are conducive to the rational use of medical resources and reduce the economic burden of patients. The lighter economic burden is a principal factor for patients to actively choose outpatient thyroidectomy (64, 65). Data from one study show that patients with a 15.5% reduction in patient costs for outpatient thyroidectomy (66). A UK study found that each outpatient saves an average of €711 compared to inpatient costs (67). A French study showed that the cost of an outpatient thyroidectomy was $7,222, compared to $22,532 in an inpatient setting. If this group of patients was all transferred to outpatient surgery, it would save $63.6 million, which means that outpatient thyroidectomy can save about two-thirds of the cost (41).

One of the important indicators and key factors for the success of outpatient thyroidectomy is the patient’s quality of life (QoL) and satisfaction (10). Patients can return to daily life faster, which is also a principal factor for patients’ satisfaction with outpatient thyroidectomy (10). In a study that included 11 studies on patient satisfaction with outpatient surgery, 6 retrospective studies were reported, 3 were retrospective analyses of prospectively collected data, and 2 were prospective studies. It found that outpatient thyroidectomy patients were found to have a higher sense of security and higher satisfaction after discharge. Patients can get adequate home support from their family members; most patients are satisfied after a thyroidectomy on the same day (10). Careful patient selection and assurance of the patient quality of life are the keys to patient satisfaction.

## Discussion

In recent decades, more and more patients and medical institutions favor overnight and outpatient thyroidectomy, and the proportion of them is increasing year by year. Multiple studies around the world all indicate that overnight and outpatient operations have a shorter hospital stay and economic benefits. Meanwhile, there is no significant difference in the incidence of complications between them and inpatient thyroidectomy. However, overnight and outpatient thyroidectomy are still facing challenges (68).

With the development of medical technology, the safety of overnight or outpatient thyroidectomy continues to improve through smaller incisions, better hemostasis, better anesthesia, intraoperative neuromonitoring, and rapid postoperative detection of PTH. While there is no statistical difference in the incidence of complications between overnight, outpatient thyroidectomy and inpatient thyroidectomy, there are still some differences in their management of complication. Compared with inpatient thyroidectomy, overnight or outpatient thyroidectomy requires more human support, and caregivers need to spend more time and energy at home to care for patients and monitor complications, which puts forward higher requirements for family caregivers. If the hospital and doctors are strictly controlled the selection of patients and indications, the complication of overnight or outpatient thyroidectomy can be controlled (51, 69).

Overnight thyroidectomy is mature at present, but there have been advances in recent years. From the perspective of the surgery approaches, there are more surgical approaches have gradually appeared in overnight thyroidectomy in recent years such as TOETVA and ECTBA. However, there are no studies of new surgical approaches currently in outpatient thyroidectomy. Outpatient thyroidectomy is still dominated by unilateral thyroidectomy, with bilateral thyroidectomy and central neck dissection accounting for a relatively low proportion. On the one hand, more research is needed to confirm the safety and economic benefits of these novel surgical approaches. On the other hand, we also look forward to more surgical approaches in overnight and outpatient thyroidectomy, as well as the possibility of greater surgery extent, looking for the best balance between surgical scope and complication rates.

From an economic point of view, overnight or outpatient thyroidectomy has certain advantages over inpatient thyroidectomy after careful patient selection. Medical institutions should carefully select patients, continuously improve the quality of medical care, and continuously improve measures to reduce readmission rates (70). At present, there are few randomized controlled trials on overnight and outpatient surgery. Therefore, it is hoped that more research will further explore innovative safety and cost-effective surgical models. Furthermore, patient’s mental health also needs to be considered. patients will have an inherent sense of security when they are in the hospital. Due to the shorter observation time than inpatient surgery, patients are more anxious, and their stress levels have increased (15, 16). Therefore, for overnight and outpatient thyroidectomy medical staff should pay more attention to the psychological counseling of patients, which can further improve the satisfaction of patients. We also expect more research to focus on patients’ mental health and find appropriate psychological interventions to help patients reduce negative emotions after thyroidectomy.

Currently, overnight and outpatient thyroidectomy has not been widely performed in many developing countries, especially outpatient thyroidectomy, due to safety concerns. The author’s country has been performing overnight thyroidectomy for many years but has not accepted outpatient thyroidectomy for safety reasons. The patient may not be able to reach the nearest medical institution in time due to reasons such as traffic or distance, which will increase the risk to the patient’s life. In addition, the level of primary medical care is lagging in developing countries, and the medical staff in primary medical care is not experienced enough. Sometimes even if the patient arrives at the community hospital, the doctors in the community hospital may not be able to deal with the postoperative complications in the correct manner, which is also a crucial factor limiting the development of overnight or outpatient thyroidectomy. Thus, for these above reasons there is still a long way to go in the spread and implementation of overnight and outpatient surgery worldwide, especially outpatient thyroidectomy.

In conclusion, our review discussed and summarized recent advances in overnight and outpatient thyroidectomy and further emphasized that overnight and outpatient thyroidectomy are safe and cost-effective. But it must be practice with strict patient selection criteria and focus on the patient and caregiver’s training and mental management. We would still like to see more randomized controlled trials and more surgical approaches in overnight or outpatient thyroidectomy. We believe that, with the great improvement of basic medical care in the world and the establishment of indications and procedures for the standardization of overnight and outpatient thyroidectomy, the overnight and outpatient thyroidectomy will be greatly promoted worldwide.

## Author contributions

All authors made substantive intellectual contributions to this study. XL and FX conceived of the design of the study. DS, ZZ prepared the manuscript. XL and FX edited the manuscript. All authors read and approved the final manuscript. All authors contributed to the article and approved the submitted version.
